# Viral influence on chemical communication in eusocial insects: insights and emerging research priorities

**DOI:** 10.1093/jisesa/ieag063

**Published:** 2026-07-22

**Authors:** Bashiru Adams, Natraj Krishnan, Esmaeil Amiri, Jian Chen

**Affiliations:** Delta Research and Extension Center, Mississippi State University, Stoneville, MS, USA; Biological Control of Pests Research Unit, USDA-ARS, Stoneville, MS, USA; Department of Agricultural Science and Plant Protection, Mississippi State University, Mississippi State, MS, USA; Delta Research and Extension Center, Mississippi State University, Stoneville, MS, USA; Department of Agricultural Science and Plant Protection, Mississippi State University, Mississippi State, MS, USA; Biological Control of Pests Research Unit, USDA-ARS, Stoneville, MS, USA

**Keywords:** Eusocial insects *Apis mellifera*, *Solenopsis invicta*, exocrine gland, pheromone, behavior

## Abstract

Chemical communication in eusocial insects is crucial for colony survival and adaptation to diverse environmental conditions, contributing significantly to their evolutionary success. Pheromones, produced mainly by exocrine glands, serve as the primary means of communication among colony members. They coordinate complex social behaviors, including foraging, queen recognition, brood care, alarm signaling, and colony defense. Proper pheromone function is therefore essential for maintaining colony cohesion and efficiency. However, pathogens, particularly viruses, can influence pheromone-mediated communication through 2 main mechanisms: by altering pheromone production or by impairing pheromone perception. The impacts of viruses on exocrine glands and pheromone production have been well documented in several nonsocial insect species. Although evidence is growing for the effects of viruses on pheromone-mediated behaviors in eusocial insects, the underlying mechanisms linking viruses and pheromone signaling remain poorly understood. Here, we review current knowledge on how viral infections influence chemical communication in 2 ecologically important, eusocial insects: the honey bee (*Apis mellifera* Linnaeus, 1758), a nectar and pollen feeder, valued for its pollination services and honey production, and the fire ant (*Solenopsis invicta* Buren, 1972), an omnivore and notorious invasive pest. We further explore the ecological implications of virus–eusocial insect interactions and discuss their consequences for pollinator and pest management. Additionally, we identify knowledge gaps and highlight future research directions that could enhance our understanding of virus–pheromone dependencies. By examining these complex interactions through a chemical ecology perspective, we aim to advance the understanding of viral ecology and contribute to the development of management strategies that improve honey bee health and enable sustainable fire ant control.

## Introduction

Chemical communication is vital for survival in organisms, especially insects (Moyano et al. 2023, Adams et al. 2024, 2025). Insects rely on chemical signals in their environment for various purposes, including locating food sources, avoiding natural enemies, and coordinating conspecific behaviors such as mating, aggregation, and defense (Scheidler et al. 2015, Chen et al. 2022). In social insect species, such as ants, bees, and termites, chemical communication becomes even more complex and indispensable, as it underpins the extraordinary level of social organization and cooperation observed within colonies (Ali and Morgan 1990, Ayasse et al. 2001, Trhlin and Rajchard 2011). The diverse pheromones produced by different castes, and their synchronized effects, play an essential role in coordinating individual and colony-level processes (Fletcher and Blum 1981, Vargo and Hulsey 2000, Le Conte and Hefetz 2008). Pheromones trigger specific behavioral or physiological responses within a colony, facilitating a wide array of critical colony functions, such as nestmate recognition, brood care, foraging, alarm signaling, caste regulation, and the maintenance of queen dominance (Le Conte et al. 2001, Fan et al. 2010, Starkey and Tamborindeguy 2023).

Insects, like other organisms, are vulnerable to infection by diverse pathogens. In addition to causing disease, pathogen infections can alter pheromone production and chemical communication by impairing the normal functioning of insect glands, especially exocrine glands, with potential consequences for host behavior manipulation and pathogen transmission dynamics (Prompiboon et al. 2010, Guerra et al. 2015). Exocrine glands are specialized organs that secrete chemicals outside the body through ducts, playing a key role in chemical communication in insects. In honey bees, the cephalic salivary glands are one of the few major sites where hydrocarbons are stored, and these compounds function as key cues for nestmate recognition (Richard et al. 2012, Martin et al. 2018), whereas in ants, the postpharyngeal gland is the major exocrine gland responsible for hydrocarbon storage (Kaib et al. 2000). Several studies have demonstrated that viral infections can negatively affect exocrine glands across diverse insect species. For example, *Glossina pallidipes* salivary gland hypertrophy virus infects the tsetse fly (*G. pallidipes*) salivary glands, causing hypertrophy and dysfunction that, in turn, reduce feeding efficiency and compromise the transmission of other pathogens (Guerra et al. 2015). Likewise, infection of the housefly (*Musca domestica*) salivary glands by the *M. domestica* salivary gland hypertrophy virus induces hypertrophy, leading to reduced reproductive capacity and altered behavior, ultimately impacting population dynamics (Prompiboon et al. 2010). Interestingly, in the Dufour’s and venom glands of some parasitic wasps, such as *Biosteres longicaudatus* and *Leptopilina boulardi*, virus-like particles and polydnaviruses play roles in facilitating the protection and development of the wasp’s eggs within their prey hosts (Lawrence and Akin 1990, Dupas et al. 2008, Bousquet et al. 2012). These examples collectively illustrate how viruses can target insect exocrine glands, resulting in a range of physiological and behavioral consequences for their hosts.

Viral infections can influence the production and perception of chemical signals, key processes that are central to insect chemical communication (Gao et al. 2023). For instance, infection by the rod-shaped enveloped *Helicoverpa zea* nudivirus 2 virus (Hz-2v) alters sex pheromone production in female corn earworm moths (*Helicoverpa zea*), a change associated with enhanced attraction of males (Burand et al. 2005). Similarly, infection of the invasive fall armyworm (*Spodoptera frugiperda*) by the baculovirus *Autographa californica* multiple nucleopolyhedrovirus suppresses the genes *Desaturase1* (*Desat1*) and *bond*, which are known to play roles in pheromone production and perception in other insect species (Pantha et al. 2021). Moreover, Drosophila C virus infection in the fruit fly (*Drosophila melanogaster*) induces the expression of pherokine-2 (Phk-2), a protein related to OS-D/A10 (Phk-1) that functions as a putative odor/pheromone-binding protein expressed in the antennae (Sabatier et al. 2003). These findings suggest that viruses may modulate chemical communication; however, this phenomenon has been poorly investigated, especially in social insect species.

Social insects are inherently prone to viral infections (Schmid-Hempel 1995). Their vulnerability largely stems from colony living, which involves high humidity in the nest, dense populations of genetically related individuals, frequent interactions among different developmental stages, and extensive body contact and fluid exchange factors that facilitate rapid pathogen transmission among colony members (Elizalde et al. 2020, Abbot 2022). Acting across multiple biological levels, viral infections can induce physiological, genetic, and behavioral changes (Galbraith et al. 2015, Hsu et al. 2018, Lemanski et al. 2021), thereby compromising their health and social organization (Chen et al. 2014, Amiri et al. 2017, Kim et al. 2019). Viruses are also a major factor contributing to colony mortality, though their impacts are better characterized in honey bees than in ants or termites (McMenamin and Genersch 2015, Lamas and Evans 2024, Tiritelli et al. 2025). This colony mortality may, in part, result from the disruption of precise communication among colony members (Geffre et al. 2020, Valles 2023), as many colony-level behaviors rely on tightly coordinated complex physiological processes (Ali and Morgan 1990, Friedman et al. 2020).

Although a wealth of evidence shows that viruses adversely affect social insects, their effects are not entirely explained by acute mortality, subtle or overt pathogenic symptoms, suggesting that viruses may affect other aspects of insect biology, such as chemical communication. Chemical signals often remain reliable indicators of altered physiological status (Moyano et al. 2023). Viral infections commonly alter host physiology by activating immune pathways, which can modify cuticular hydrocarbon profiles and directly affect exocrine glands, thereby altering pheromone-related chemical profiles and impairing signal biosynthesis (Geffre et al. 2020, Pant et al. 2021). Viral infections that disrupt the physiology of social insects may also impair pheromone-mediated communication, potentially exerting profound and multifaceted effects on these systems (Orlova et al. 2023, McAfee et al. 2025). However, the mechanisms linking viruses to pheromone-mediated communication remain poorly understood in social insect species.

Here, we review current knowledge on the impact of viruses on chemical communication in two of the most extensively studied social insects, honey bees and fire ants, which represent opposite extremes in their ecological and economic significance to humans. Honey bees (*Apis* spp.) include several species, among which the Western honey bee (*Apis mellifera* Linnaeus) is the most extensively managed for apiculture purposes and agricultural pollination services (Garrido and Nanetti 2019). Through widespread human-mediated translocation, *A. mellifera* (hereafter referred to as the honey bee) has spread far beyond its native range and is now present across nearly all climatically suitable and inhabited regions of the world (Garrido and Nanetti 2019), underscoring the need to identify sustainable strategies to protect bee health. In contrast, invasive fire ants (*Solenopsis* spp.) are notorious pests with significant ecological, agricultural, and medical consequences (Calcaterra et al. 2008, Wen et al. 2021). They negatively impact agricultural, urban, and natural habitats (Williams et al. 2001, Calcaterra et al. 2008). The ongoing global expansion of fire ants, facilitated primarily by human activities, through international trade and transportation (King et al. 2009, Ascunce et al. 2011), exposes them to encounter novel environmental stressors and pathogens, some of which may possess effective control potential for use in biological control programs (Garrido and Nanetti 2019, Holmes and Johnston 2022). In this review, we discuss the ecological consequences of virus–eusocial insect interactions and their implications for pest and pollinator management strategies. Finally, we highlight key knowledge gaps and suggest future research directions to improve honey bee health and explore the potential application of viruses in fire ant management.

## Overview of Viruses Associated With Honey Bees and Fire Ants

Viruses are ubiquitous in nature and can infect a wide range of organisms, including social insects such as honey bees and fire ants. RNA viruses are the most prevalent viral group in these insects, infecting individuals across all castes and developmental stages (Valles 2012, Amiri et al. 2021, Ullah et al. 2021, Li et al. 2024). In honey bees, viruses represent the largest class of infecting pathogens, with over 50 different viruses described and likely many more yet to be discovered (McMenamin and Flenniken 2018, Beaurepaire et al. 2020, Deboutte et al. 2024, Kadlečková et al. 2024). The most studied viruses that cause symptomatic diseases and colony weakening in honey bees are positive-sense, single-stranded RNA (+RNA) viruses (Chen et al. 2004, McMenamin and Flenniken 2018, Grozinger and Flenniken 2019, Amiri et al. 2021). They can be classified into the families *Dicistroviridae* (black queen cell virus [BQCV], acute bee paralysis virus [ABPV], Israeli acute paralysis virus [IAPV], and Kashmir bee virus [KBV]), *Iflaviridae* (deformed wing virus [DWV], slow bee paralysis virus [SBPV], sacbrood virus [SBV]), and unassigned families (chronic bee paralysis virus [CBPV], and Lake Sinai viruses [LSVs]). It is also worth noting that DNA viruses have been identified in honey bees (Kadlečková et al. 2024). The *Apis mellifera* filamentous virus is characterized as an enveloped double-stranded DNA (dsDNA) virus (Bailey et al. 1981, Sitaropoulou et al. 1989, Gauthier et al. 2015). More recently, researchers discovered another dsDNA virus, called *Apis mellifera* filamentous-like virus, using metagenomic sequencing of honey bees collected in the Czech Republic (Kadlečková et al. 2024). The development of high-throughput sequencing technologies and bioinformatic tools has greatly facilitated the identification and characterization of novel viruses in honey bees (McMenamin and Flenniken 2018). These newly discovered viruses include a wide range of types, such as positive-sense, single-stranded RNA (+ssRNA) viruses, negative-sense, single-stranded RNA (−ssRNA) viruses, and DNA viruses, including those that belong to the *Tymoviridae, Secoviridae, Nodaviridae, Flaviviridae, Bunyaviridae, Iflaviridae*, *Rhabdoviridae*, and *Parvoviridae* families (Runckel et al. 2011, McMenamin and Flenniken 2018, Kraberger et al. 2019, Daughenbaugh et al. 2021, Kadlečková et al. 2024). Many of these viruses have only recently been discovered, therefore, information regarding their pathology, mode of transmission, and effects on individual bees or colonies remains limited (McMenamin and Flenniken 2018).

To date, approximately 22 viruses have been identified worldwide in fire ants (Oliver et al. 2025, Valles et al. 2025), predominantly in the red imported fire ant (*S. invicta*) (Xavier et al. 2021, Li et al. 2024). More than half of these viruses are +ssRNA, although other viruses, including −ssRNA, double-stranded RNA (ds-RNA), and dsDNA, have also been found in fire ants (Baty et al. 2020). For example, ssRNA viruses from the *Dicistroviridae* family, including *Solenopsis invicta* viruses (SINV-1, SINV-2, up to SINV-17), 1 ds-RNA virus (*Solenopsis* midden virus), *Nylanderia fulva* virus 1, and DWV (Table 1) are the most common viruses of fire ants (Valles and Rivers 2019, Xavier et al. 2021, Miles et al. 2023, 2024). A recent study has also reported 15 new viruses belonging to the families *Iflaviridae*, *Polycipiviridae*, *Dicistroviridae*, and *Parvoviridae* in populations of fire ants in China (Table 1, Li et al. 2024). Viruses in the family *Solinviviridae* (SINVs) are considered the most abundant natural enemies of fire ants (Valles and Rivers 2019, Baty et al. 2020). Ecological interactions between honey bees and fire ants, such as robbing pollen or honey from bee hives, scavenging dead adult bees, or cohabitation, can facilitate the horizontal transmission of these viruses from honey bees to fire ants (Payne et al. 2020, Valizadeh et al. 2025). As a result, several honey bee-associated viruses, including DWV, BQCV, ABPV, KBV, and IAPV, have also been detected in fire ants (Table 1, Baty et al. 2020, Payne et al. 2020, Tiritelli et al. 2025).

**Table 1.** List of some important viruses reported in honeybees and fire ants, caste and developmental stages affected, and body parts or tissues targeted

Virus	Honey bee	Fire ant
**Deformed wing virus**	Infects all castes and developmental stages and targets multiple tissues, especially wings and brain (Kim et al. 2019, Amiri et al. 2020b, Silva et al. 2021)	Detected across all major castes in multiple tissues, especially wings (Hsu et al. 2018, Baty et al. 2020, Payne et al. 2020, Miles et al. 2024)
**Sacbrood virus**	Attacks primarily brood, especially young larvae, but can infect adults. It targets gut and epithelial tissues in brood, disrupting metamorphoses, but reproductive and glandular tissues are targeted in adults (Amiri et al. 2020a, Beaurepaire et al. 2020, Durand et al. 2026)	—
**Israel acute paralysis virus**	Infect all castes and developmental stages, targeting the nervous system, gut epithelia, trachea, and hypopharyngeal gland (Chen et al. 2014, Amiri et al. 2019)	All developmental stages are affected, targeting every part of the body (Baty et al. 2020, Payne et al. 2020, Valizadeh et al. 2025)
**Slow bee paralysis virus**	Systemic virus of adult bees, affecting the nervous system and hemolymph (McMenamin and Flenniken 2018, Beaurepaire et al. 2020)	—
**Black queen cell virus**	It affects all castes and developmental stages, especially queen larvae and pupae. It targets the gut, fat body, glands, and hemolymph (Spurny et al. 2017, Al Naggar and Paxton 2020)	It is detected in fire ants, but there is no clear evidence of tissue infection and replication in fire ants (Baty et al. 2020, Tiritelli et al. 2025)
**Acute bee paralysis virus**	Affects all castes and developmental stages, targeting hemolymph and the nervous system (Azzami et al. 2012, Ullah et al. 2021)	It is detected in fire ants, but there is no clear evidence of tissue infection and replication in fire ants (Schmid-Hempel 1995, Baty et al. 2020, Tiritelli et al. 2025)
***Apis mellifera* filamentous virus**	Affects all castes and developmental stages, and it targets all tissues, including the brain and abdominal organs (Bailey et al. 1981, Sitaropoulou et al. 1989, Sébastien et al. 2015)	—
**Kashmir bee virus**	Affects all castes and developmental stages, and it targets all body tissues (Beaurepaire et al. 2020, Ullah et al. 2021)	Detected in fire ants, but tissue tropism, caste range, and pathogenicity remain largely unknown (Baty et al. 2020, Tiritelli et al. 2025)
**Bee macula-like virus**	Infects adult workers and pupae and is widespread at the colony level, with likely but not fully mapped presence in queens and drones. It targets the whole-body, but its precise caste range and tissue tropism are not yet fully characterized (Ravoet et al. 2015, McMenamin and Flenniken 2018, Beaurepaire et al. 2020)	—
**Aphid lethal paralysis virus**	Present and replicates at the whole-bee level; caste-, stage-, and tissue-specific tropisms in honey bees remain largely unresolved (Ravoet et al. 2015, Baty et al. 2020)	—
**Chronic bee paralysis virus**	Affects adult workers, but also capable of infecting queens, establishing a systemic infection with the highest viral loads in the brain and hindgut and lower but detectable levels in the fat body, trachea, midgut, and thorax (Amiri et al. 2020b, Deng et al. 2024)	—
**Lake sinai virus**	Infects adults but can affect the brood. It targets the gut and abdomen, and lower levels, head, and thorax (Ravoet et al. 2015, McMenamin and Flenniken 2018, Daughenbaugh et al. 2021)	—
**Tobacco ringspot virus**	It affects all castes and developmental stages, including eggs. Targets multiple body parts (head, thorax, abdomen, legs, wings) (Li et al. 2014, Beaurepaire et al. 2020)	—
**Moku virus**	Generally, attack adults, but their caste range, developmental-stage specificity, and tissue tropism within honey bee bodies are still largely unknown (Mordecai et al. 2016)	—
***Solenopsis invicta* virus**	—	It affects all castes and developmental stages while targeting all tissues, but prefers the alimentary canal (especially the midgut and hindgut) (Hashimoto and Valles 2007, 2008, Valles and Hashimoto 2009)
***Solenopsis midden* virus**	—	Associated with workers and brood; its true host range within castes and developmental stages, and its tissue tropism have not yet been characterized (Baty et al. 2020, Holmes and Johnston 2023)
***Nylanderia fulva* virus**	—	Infects larvae, pupae, workers, and queens, but tissue tropism has not yet been characterized (Baty et al. 2020, Payne et al. 2020, Holmes and Johnston 2022)
***Solenopsis invicta* densovirus**	—	Infects workers and brood, but its organ-level tissue tropism has not yet been characterized (Baty et al. 2020, Li et al. 2024)
**Guangdong Polycipiviridae ant virus 1**	—	Beyond its genomic characterization, its caste and developmental-stage range and tissue tropism are currently unknown (Li et al. 2024)
**Guangdong Dicistroviridae ant virus 2-3**	—	Beyond its genomic characterization, its caste and developmental-stage range and tissue tropism are currently unknown (Li et al. 2024)
**Guangdong Iflaviridae ant virus 4-9**	—	Beyond its genomic characterization, its caste and developmental-stage range and tissue tropism are currently unknown (Li et al. 2024)
**Guangdong Parvoviridae ant virus 10**	—	Beyond its genomic characterization, its caste and developmental-stage range and tissue tropism are currently unknown (Li et al. 2024)
**Guangdong ant virus 11-15**	—	Beyond its genomic characterization, its caste and developmental-stage range and tissue tropism are currently unknown (Li et al. 2024)

The symbol (—) indicates the absence of the virus.

## Virus Transmission in Honey Bees and Fire Ants

**Fig. 1.**
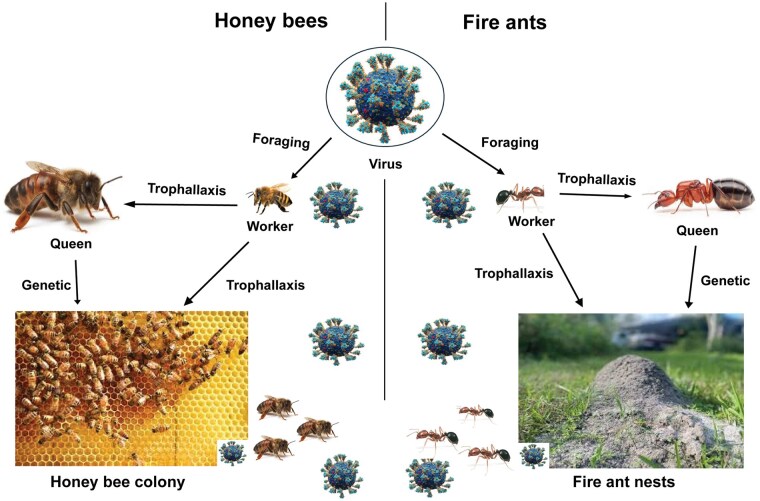
Modes of virus transmission in honey bees and fire ants. The diagram shows virus transmission pathways in a honey bee colony on the left and a fire ant nest on the right. Horizontal transmission occurs from food sources to foraging workers and is subsequently transmitted by workers to queens and other nestmates through trophallaxis, as shown with horizontal arrows in honey bee colonies and fire ant nests. Vertical transmission occurs when infected queens transfer the infection to their offspring (eggs and brood), as shown in vertical arrows in both honey bees and fire ants.

Understanding transmission routes and the directionality of viral spread is a crucial first step in determining the effects and epidemiology of a given pathogen, because transmission routes directly affect the prevalence and virulence of viruses (Fries and Camazine 2001, Alizon et al. 2009, Martin et al. 2012). In complex social colony structures, viruses can spread through various transmission routes: horizontally via trophallaxis or food exchange, glandular secretions, direct body contact, and vectors, or venereally through mating (Fries and Camazine 2001, Chen et al. 2006, Yañez et al. 2020) and vertically through eggs and stored sperm, as shown in Fig. 1 (de Miranda et al. 2010, Ravoet et al. 2015, Yañez et al. 2020).

In horizontal transmission, the main transmission route is through the oral–fecal pathway from infected food sources, such as flowers, nectar, or pollen (Singh et al. 2010, Yañez et al. 2020), and broods may also acquire viruses from workers through trophallaxis, ie direct transfer of food or other fluids between members of a colony, as shown in Fig. 1 (Pie et al. 2004). Body contact is also known to help virus transmission among nestmates (Fig. 1, Naug and Camazine 2002, Pie et al. 2004, Yañez et al. 2020). Moreover, retinue workers during queen tending may transmit viruses to the queen (Amiri et al. 2019). Pupal cannibalism by worker honey bees is also a horizontal pathway that contributes to the spread of deformed wing virus (Posada-Florez et al. 2021). It is also a common behavior in fire ant colonies, as sometimes workers execute and eat female alates, attributed to nutritional stress (Dai et al. 2025). However, there is no evidence that cannibalism causes disease transmission in fire ants. Venereal transmission (the spread of viruses from drones to queens during mating) is a form of horizontal transmission in eusocial insects (Yañez et al. 2020).

Vector-mediated virus transmission is another form of horizontal transmission in eusocial insects. Many economically important viruses affecting eusocial insects, especially honey bees, are associated with parasitic infestations (Al Naggar and Paxton 2020, Ryabov et al. 2022). For instance, *Varroa* mites (*Varroa destructor*), an important parasite of honey bees, act as both a mechanical and biological vector and transmit several viruses, including DWV, ABPV, IAPV, SBPV, KBV, bee macula-like virus (MLV), and *Varroa* tymo-like virus (Beaurepaire et al. 2020, Ryabov et al. 2022). However, evidence of vector-borne virus transmission in fire ants is limited.

Vertical virus transmission in eusocial insects may occur through eggs (transovum or/and transovarial) when virus-infected queens transfer viruses to their offspring (Fig. 1). For example, in honey bees, viruses, including DWV, SBV, aphid lethal paralysis virus, LSVs, and *V. destructor* MLV, are transmitted from infected queens to their offspring (Ravoet et al. 2015, Amiri et al. 2020a). In fire ants, distinguishing between horizontal and vertical transmission is challenging due to the complex interactions among colony members (Xavier et al. 2021). Evidence of vertical transmission of viruses in fire ants is very scarce, but SINV-2 and IAPV are vertically transmitted in *S. invicta* (Hashimoto and Valles 2008, Valizadeh et al. 2025), suggesting the possibility of transferring other viruses via this transmission route (Fig. 1).

## Impact of Virus Infections on Honey Bees and Fire Ants

Viruses are among the most important, yet least understood, pathogens affecting social insects, with direct impacts on growth, physiology, behavior, morphology, and reproduction (Table 1, Manley et al. 2015, Geffre et al. 2020). Viral coinfection frequently occurs in social insects, amplifying the virus’s impact and worsening the effects of individual infections, which must be considered (Chen et al. 2004, Durand et al. 2023, 2026). In response to viral infections, eusocial insects, including honey bees and fire ants, exhibit a range of physiological and behavioral responses (Hashimoto and Valles 2008, Amiri et al. 2020a, Miles et al. 2024). Virus-induced physiological alterations may include deformity of body parts, neurological impairments, weight loss, immune responses, and shifts in microbial community (Brutscher et al. 2016, Grozinger and Flenniken 2019, Miles et al. 2023). For instance, DWV (Shah et al. 2009, Ferreira et al. 2025) and CBPV (Olivier et al. 2008) have been shown to affect the mushroom bodies and brain of honey bees, whereas IAPV and ABPV target the brain and central nervous system (Li et al. 2013b, Fulton et al. 2025). Likewise, DWV targets the wings of fire ants (Miles et al. 2023, 2024), whereas SINV-1 affects the midgut (Hsu et al. 2018). These changes, in turn, influence key social behaviors such as foraging, aggression, nesting, territory defense, brood rearing, and colony organization in both honey bees (Fujiyuki et al. 2005, Shah et al. 2009, Coulon et al. 2020, Geffre et al. 2020, Taylor and Dolezal 2024, Fulton et al. 2025) and fire ants (Hsu et al. 2018, Miles et al. 2023, 2024). However, less is known about how viral infections influence chemical communications, the cornerstone of social organization in social species. Viral infections that alter host physiology may negatively impact the synthesis, release, or perception of the chemical signals, potentially leading to changes in social cohesion and colony efficiency. Understanding the mechanistic links between viral infection and chemical communication is therefore critical for elucidating how viral pathogens modulate social insect behavior and colony dynamics.

### Exocrine Glands and Pheromone Production in Honey Bees and Fire Ants

**Fig. 2.**
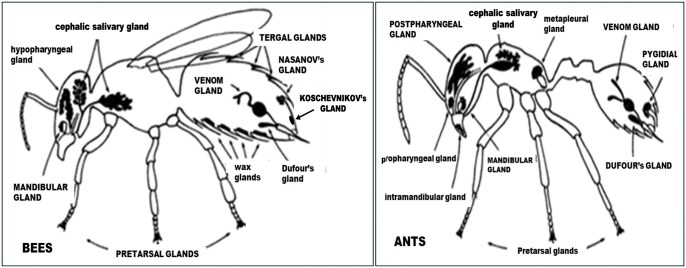
Examples of commonly found exocrine glands in 2 social insect groups (bees and ants) associated with pheromones that mediate chemical interactions. Glands with a pheromonal function are indicated in capital letters (Billen and Morgan 1998). The diagram shows the location of exocrine glands in the head, thorax, abdomen, and legs of honey bees on the left and fire ants on the right.

Chemical communication is essential for honey bee and fire ant colonies, supporting almost all aspects of their organization, cooperation, and survival (Ali and Morgan 1990, Ayasse et al. 2001, Fan et al. 2010, Trhlin and Rajchard 2011, Manfredini et al. 2016, Yan and Liebig 2021). They use pheromones to transmit vital information, such as coordinating activities, signaling dangers, maintaining social hierarchies, and ensuring smooth functioning among thousands of members (Table 2, Ali and Morgan 1990, Ayasse et al. 2001, Trhlin and Rajchard 2011). Pheromones play crucial and diverse roles in the chemical communication of eusocial insects (Leonhardt et al. 2016, Ge et al. 2020, Yan and Liebig 2021). Pheromones are categorized into releaser (elicit immediate behavior) and primer (induce longer term physiological changes) pheromones (Regnier and Law 1968, Ali and Morgan 1990). Since eusocial insects possess numerous pheromone-producing exocrine glands across all castes (queens and workers) and drones, these glands are distributed throughout their bodies, particularly in regions directly linked to their specific roles (Fig. 2, Table 2). A classic example is the queen-released chemical signals from specific glands in honey bees and fire ants, which influence other castes’ behavior, development, and physiology, especially the workers (Fletcher and Blum 1981, Vargo 1992, Vander Meer 1998, Vargo and Hulsey 2000, Fan et al. 2010, Orlova et al. 2023).

**Table 2.** Examples of studies that reported major and minor chemical compounds/pheromones from the exocrine glands of honeybees and fire ants

Exocrine gland and associated pheromones	Chemical class	Developmental stages and castes that produce pheromone, and their functions	
		Honey bees	Fire ants
Mandibular gland			
**2-heptanone**	Methyl ketone	Produced by worker honey bees, it functions as an alarm/defensive secretion and local anesthetic against small arthropods (Boch et al. 1962)	—
**2-ethyl-3,6(5)-dimethyl pyrazine**	Alkyl pyrazine	—	Produced by worker ants, it is a component of alarm pheromone that rapidly activates workers, increasing aggression, recruitment, and defensive responses around a disturbance source (Chen 2023, Noël et al. 2023)
**9-hydroxy-(*E*)-2-decenoic acid**	Fatty acid	It is a key component of the queen retinue pheromone, and is responsible for many of the primer and releaser effects on worker behavior and reproduction (Butler et al. 1962, Ali and Morgan 1990, Kocher and Grozinger 2011)	—
**9-oxo-(*E*)-2-decenoic acid**	Fatty acid	It is a key component of the queen retinue pheromone, and is responsible for many of the primer and releaser effects on worker behavior and reproduction (Butler et al. 1962, Ali and Morgan 1990, Keeling et al. 2003)	—
**10-hydroxydecanoic acid**	Fatty acid	Produced in the mandibular gland of royal jelly-producing workers, and it is associated with jelly for larvae and queens (Ali and Morgan 1990, Plettner et al. 1995, Walsh et al. 2020)	—
**4-hydroxy-3-methoxyphenylethanol**	Fatty acid	Produced by mature queens, it is responsible for eliciting worker retinue behavior (Ali and Morgan 1990, Fan et al. 2010, Niño et al. 2013)	—
**Methyl *p*-hydroxybenzoate**	Fatty acid	Produced by mature queens, it is responsible for eliciting worker retinue behavior (Butler et al. 1962, Kocher and Grozinger 2011)	—
**Palmitic acid**	Fatty acid	Produced by queens, and it contributes to the queen recognition cues (Trhlin and Rajchard 2011)	—
**Oleic acid**	Fatty acid	Associated with queens’ and workers’ secretions and brood. Functions both as a conserved ‘death cue’ associated with brood removal and as a common fatty acid-derived lipid, linked to nutritional secretions (Trhlin and Rajchard 2011, Martin et al. 2018, McAfee et al. 2025)	
**Stearic acid**	Fatty acid	Associated with worker bees and royal jelly and serves as the metabolic precursor for the caste-specific mandibular gland decenoic acids (Trhlin and Rajchard 2011, Wu et al. 2017)	—
**Behenyl alcohol (1-docosanol)**	Fatty alcohol	Produced in the queen mandibular gland, it is a minor constituent of the queen retinue pheromone blend (Keeling et al. 2003)	—
**Dufour’s gland**			
**Tetradecyl dodecanoate**	Wax ester	Produced by queens and laying workers and contribute to modifying the egg discriminatory behavior of worker bees (Katzav-Gozansky et al. 2001)	—
**Tetradecyl (*Z*)-9-tetradecenoate**	Wax ester	Produced by queens and laying workers and contribute to modifying the egg discriminatory behavior of worker bees (Katzav-Gozansky et al. 2001)	—
**Tetradecyl tetradecanoate**	Wax ester	Produced by queens and laying workers and contribute to modifying the egg discriminatory behavior of worker bees (Katzav-Gozansky et al. 2001)	—
**Tetradecyl (*Z*)-11-hexadecenoate**	Wax ester	Produced by queens and laying workers and contribute to modifying the egg discriminatory behavior of worker bees (Katzav-Gozansky et al. 2001, Martin et al. 2002)	—
**Tetradecyl (*Z*)-9-hexadecenoate**	Wax ester	Produced by queens and laying workers and contribute to modifying the egg discriminatory behavior of worker bees (Katzav-Gozansky et al. 2001, Martin et al. 2002)	—
**Tetradecyl hexadecanoate**	Wax ester	Produced by queens and laying workers and contribute to modifying the egg discriminatory behavior of worker bees (Katzav-Gozansky et al. 2001, Martin et al. 2002)	—
**Hexadecyl tetradecanoate**	Wax ester	Produced by queens and laying workers and contribute to modifying the egg discriminatory behavior of worker bees (Katzav-Gozansky et al. 2001, Martin et al. 2002)	—
**Hexadecyl octadecanoate**	Wax ester	Produced by queens and laying workers and contribute to modifying the egg discriminatory behavior of worker bees (Martin et al. 2002)	—
**Hexadececyl octadecenoate**	Wax ester	Produced by queens and laying workers and contribute to modifying the egg discriminatory behavior of worker bees (Martin et al. 2002)	—
**Tetradecyl (*Z*)-9-octadecenoate**	Wax ester	Produced by queens and laying workers and contribute to modifying the egg discriminatory behavior of worker bees (Katzav-Gozansky et al. 2001, Martin et al. 2002)	—
**(*Z*)-9-hexadecenyl hexadecanoate**	Wax ester	Produced by queens and laying workers and contribute to modifying the egg discriminatory behavior of worker bees (Katzav-Gozansky et al. 2001, Martin et al. 2002)	—
**Hexadecyl hexadecanoate**	Wax ester	Produced by queens and laying workers and contribute to modifying the egg discriminatory behavior of worker bees (Katzav-Gozansky et al. 2001, Martin et al. 2002)	—
**Octadecyl tetradecanoate**	Wax ester	Produced by queens and laying workers and contribute to modifying the egg discriminatory behavior of worker bees (Katzav-Gozansky et al. 2001, Martin et al. 2002)	—
**(*Z*)-9-hexadecenyl (*Z*)-9-octadecenoate**	Wax ester	Produced by queens and laying workers and contribute to modifying the egg discriminatory behavior of worker bees (Katzav-Gozansky et al. 2001)	—
**Octadecyl hexadecanoate**	Wax ester	Produced by queens and laying workers and contribute to modifying the egg discriminatory behavior of worker bees (Katzav-Gozansky et al. 2001)	—
**Octadecenyl octadecenoate**	Wax ester	Produced by queens and laying workers and contribute to modifying the egg discriminatory behavior of worker bees (Martin et al. 2002)	—
**Octadecyl octadecenoate**	Wax ester	Produced by queens and laying workers and contribute to modifying the egg discriminatory behavior of worker bees (Martin et al. 2002)	—
**Eicosyl octadecenoate**	Wax ester	Produced by queens and laying workers and contribute to modifying the egg discriminatory behavior of worker bees (Martin et al. 2002)	—
**Heinecosane**	Alkane	It is associated with queen/worker chemical differentiation and may contribute to the queen signal, but it has not been shown to be the primary pheromonal cue for egg recognition or policing (Katzav-Gozansky et al. 2001, Martin et al. 2002, Dor et al. 2005)	—
**Tricosane**	Alkane	It is associated with queen/worker chemical differentiation and may contribute to the queen signal, but it has not been shown to be the primary pheromonal cue for egg recognition or policing (Katzav-Gozansky et al. 2001, Martin et al. 2002, Dor et al. 2005)	—
**Tricosene**	Alkene	It is associated with queen/worker chemical differentiation and may contribute to the queen signal, but it has not been shown to be the primary pheromonal cue for egg recognition or policing (Katzav-Gozansky et al. 2001, Martin et al. 2002, Dor et al. 2005)	—
**Pentacosene**	Alkene	It is associated with queen/worker chemical differentiation and may contribute to the queen signal, but it has not been shown to be the primary pheromonal cue for egg recognition or policing (Katzav-Gozansky et al. 2001, Martin et al. 2002, Dor et al. 2005)	—
**Hexacosane**	Alkane	It is associated with queen/worker chemical differentiation and may contribute to the queen signal, but it has not been shown to be the primary pheromonal cue for egg recognition or policing (Katzav-Gozansky et al. 2001, Martin et al. 2002, Dor et al. 2005)	—
**Heptacosane**	Alkane	It is associated with queen/worker chemical differentiation and may contribute to the queen signal, but it has not been shown to be the primary pheromonal cue for egg recognition or policing (Katzav-Gozansky et al. 2001, Martin et al. 2002, Dor et al. 2005)	—
**Heptacosene**	Alkene	It is associated with queen/worker chemical differentiation and may contribute to the queen signal, but it has not been shown to be the primary pheromonal cue for egg recognition or policing (Katzav-Gozansky et al. 2001, Martin et al. 2002, Dor et al. 2005)	—
**Nonacosane**	Alkane	It is associated with queen/worker chemical differentiation and may contribute to the queen signal, but it has not been shown to be the primary pheromonal cue for egg recognition or policing (Katzav-Gozansky et al. 2001, Martin et al. 2002, Dor et al. 2005)	—
**Nonacosene**	Alkene	It is associated with queen/worker chemical differentiation and may contribute to the queen signal, but it has not been shown to be the primary pheromonal cue for egg recognition or policing (Katzav-Gozansky et al. 2001, Martin et al. 2002, Dor et al. 2005)	—
**Hentricontane**	Alkane	It is associated with queen/worker chemical differentiation and may contribute to the queen signal, but it has not been shown to be the primary pheromonal cue for egg recognition or policing (Katzav-Gozansky et al. 2001, Martin et al. 2002)	—
**Tritriacontane**	Alkane	It is associated with queen/worker chemical differentiation and may contribute to the queen signal, but it has not been shown to be the primary pheromonal cue for egg recognition or policing (Katzav-Gozansky et al. 2001, Martin et al. 2002)	—
***E*,*E*-α-farnesene**	Sesquiterpene hydrocarbon	—	Produced by worker ants and serves as a component of trail pheromone used by foragers for navigation and recruitment of conspecifics (Vander Meer et al. 1980, Chen 2024)
***Z*,*E*-α-farnesene**	Sesquiterpene hydrocarbon	—	Produced by worker ants and serves as a component of trail pheromone used by foragers for navigation and recruitment of conspecifics (Vander Meer et al. 1980, Chen 2024)
***Z*,*E*-α-homofarnesene**	Homosesquiterpene hydrocarbon	—	Produced by worker ants and serves as a component of trail pheromone used by foragers for navigation and recruitment of conspecifics (Vander Meer et al. 1980, Chen 2024)
***Z*,*Z*-α-homofarnesene**	Homosesquiterpene hydrocarbon	—	Produced by worker ants and serves as a component of trail pheromone used by foragers for navigation and recruitment of conspecifics (Vander Meer et al. 1980, Chen 2024)
**β-Farnesene**	Sesquiterpene hydrocarbon	—	Produced by worker ants and serves as a component of trail pheromone used by foragers for navigation and recruitment of conspecifics (Vander Meer et al. 1980, Chen 2024)
**Geranyl decanonate**	Monoterpene ester	—	Produced by worker ants, but the exact role is unknown (Chen 2023)
**Geranyl dodecanonate**	Monoterpene ester	—	Produced by worker ants, but the exact role is unknown (Chen 2023)
**Geranyl tetradecanonate**	Monoterpene ester	—	Produced by worker ants, but the exact role is unknown (Chen 2023)
**Geranyl hexadecanonate**	Monoterpene ester	—	Produced by worker ants, but the exact role is unknown (Chen 2023)
**Geranyl oleate**	Monoterpene ester	—	Produced by worker ants, but the exact role is unknown (Chen 2023)
**Geranyl octadecanonate**	Monoterpene ester	—	Produced by worker ants, but the exact role is unknown (Chen 2023)
**Venom/poison gland**			
**Formic acid (methanoic acid)**	Carboxylic acid	Produced in the venom gland of worker bees and occurs in the venom as an environmental treatment agent (Vander Meer 1998, Baracchi et al. 2011)	Produced in the venom gland of worker ants, and it contributes to the pain and irritation of a sting (Ali and Morgan 1990, Vander Meer 1998)
**(*E*)-2-methyl-6-nonylpiperidine**	Piperidine alkaloid	—	It is from the worker’s venom gland, and it is part of the solenopsin venom complex for causing irritation and burning pain (Chen et al. 2009, 2010, 2019, Araújo et al. 2017, Chen 2023)
**(*Z*)-2-methyl-6-nonylpiperidine**	Piperidine alkaloid	—	It is from the worker’s venom gland, and it is part of the solenopsin venom complex for causing irritation and burning pain (Chen et al. 2009, 2019, Araújo et al. 2017, Chen 2023)
**(*E*, *Z*)-2-methyl-6-undecylpiperidines**	Piperidine alkaloid	—	It is from the worker’s venom gland, and it is part of the solenopsin venom complex for causing irritation and burning pain (Chen et al. 2009, 2010, 2019, Araújo et al. 2017, Chen 2023)
**(*E*)-2-methyl-6-(undec-2-en-1-yl) piperidine**	Piperidine alkaloid	—	It is from the worker’s venom gland, and it is part of the solenopsin venom complex for causing irritation and burning pain (Chen et al. 2009, 2019, 2023, Araújo et al. 2017)
**(*Z*)-2-methyl-6-(undec-2-en-1-yl) piperidine**	Piperidine alkaloid	—	It is from the worker’s venom gland, and it is part of the solenopsin venom complex for causing irritation and burning pain (Chen et al. 2009, 2019, Araújo et al. 2017, Chen 2023)
**(*E*, *Z*)-2-methyl-6-tridecyl piperidine**	Piperidine alkaloid	—	It is from the worker’s venom gland, and it is part of the solenopsin venom complex for causing irritation and burning pain (Chen et al. 2009, 2010, 2019, Araújo et al. 2017, Chen 2023)
**(*Z*)-2-methyl-6-(tridec-3-en-1-yl) piperidine**	Piperidine alkaloid	—	It is from the worker’s venom gland, and it is part of the solenopsin venom complex for causing irritation and burning pain (Chen et al. 2009, 2019, Araújo et al. 2017, Chen 2023)
**(*Z*)-2-methyl-6-(tridec-4-en-1-yl) piperidine**	Piperidine alkaloid	—	It is from the worker’s venom gland, and it is part of the solenopsin venom complex for causing irritation and burning pain (Chen et al. 2009, 2019, Araújo et al. 2017, Chen 2023)
**(*E*)-2-methyl-6-(cis-tridec-4-en-1-yl) piperidine**	Piperidine alkaloid	—	It is from the worker’s venom gland, and it is part of the solenopsin venom complex for causing irritation and burning pain (Chen et al. 2009, 2019, Araújo et al. 2017, Chen 2023)
**(*E*)-2-methyl-6-(trans-tridec-4-en-1-yl) piperidine**	Piperidine alkaloid	—	It is from the worker’s venom gland, and it is part of the solenopsin venom complex for causing irritation and burning pain (Chen et al. 2009, 2019, Araújo et al. 2017, Chen 2023)
**(*E*)-2-methyl-6-(tridec-8-en-1-yl) piperidine**	Piperidine alkaloid	—	It is from the worker’s venom gland, and it is part of the solenopsin venom complex for causing irritation and burning pain (Chen et al. 2009, 2019, Araújo et al. 2017, Chen 2023)
**(*Z*)-2-methyl-6-(pentadec-6-en-1-yl) piperidine**	Piperidine alkaloid	—	It is from the worker’s venom gland, and it is part of the solenopsin venom complex for causing irritation and burning pain (Chen et al. 2009, 2019, Araújo et al. 2017)
**(*E*)-2-methyl-6- (cis-pentadec-6-en-1-yl) piperidine**	Piperidine alkaloid	—	It is from the worker’s venom gland, and it is part of the solenopsin venom complex for causing irritation and burning pain (Chen et al. 2009, 2019, Araújo et al. 2017, Chen 2023)
**(*E*)-2-methyl-6-(trans-pentadec-6-en-1-yl) piperidine**	Piperidine alkaloid	—	It is from the worker’s venom gland, and it is part of the solenopsin venom complex for causing irritation and burning pain (Chen et al. 2009, 2019, Araújo et al. 2017, Chen 2023)
**(*E*)-2-methyl-6-(pentadec-14-en-1-yl)piperidine**	Piperidine alkaloid	—	It is from the worker’s venom gland, and it is part of the solenopsin venom complex for causing irritation and burning pain (Chen et al. 2009, 2019, Araújo et al. 2017, Chen 2023)
**(*E*)-2-methyl-6-pentadecylpiperidine**	Piperidine alkaloid	—	It is from the worker’s venom gland, and it is part of the solenopsin venom complex for causing irritation and burning pain (Chen et al. 2009, 2019, Araújo et al. 2017, Chen 2023)
**(*E*)-2-methyl-6-(heptadec-8-en-1-yl) piperidine**	Piperidine alkaloid	—	It is from the worker’s venom gland, and it is part of the solenopsin venom complex for causing irritation and burning pain (Chen et al. 2009, 2019, Araújo et al. 2017, Chen 2023)
**(*Z*)-2-methyl-6-(heptadec-8-en-1-yl) piperidine**	Piperidine alkaloid	—	It is from the worker’s venom gland, and it is part of the solenopsin venom complex for causing irritation and burning pain (Chen et al. 2009, 2019, Araújo et al. 2017, Chen 2023)
**(*E*)-2-methyl-6-(heptadecyl)piperidine**	Piperidine alkaloid	—	It is from the worker’s venom gland, and it is part of the solenopsin venom complex for causing irritation and burning pain (Chen et al. 2009, 2019, Araújo et al. 2017, Chen 2023)
**(*Z*)-2-methyl-6-(heptadecyl)piperidine**	Piperidine alkaloid	—	It is from the worker’s venom gland, and it is part of the solenopsin venom complex for causing irritation and burning pain (Chen et al. 2009, 2019, Araújo et al. 2017, Chen 2023)
**2-methyl-6-undecyl-6-piperidene**	Piperidene alkaloid	—	It is from the worker’s venom gland, and it is part of the solenopsin venom complex for causing irritation and burning pain (Chen et al. 2009, 2019, Araújo et al. 2017, Chen 2023)
**2-methyl-6-(tridec-4-en-1-yl)-6-piperidene**	Piperidene alkaloid	—	It is from the worker’s venom gland, and it is part of the solenopsin venom complex for causing irritation and burning pain (Chen et al. 2009, 2019, Araújo et al. 2017, Chen 2023)
**2-methyl-6-tridecyl-6-piperidene**	Piperidene alkaloid	—	It is from the worker’s venom gland, and it is part of the solenopsin venom complex for causing irritation and burning pain (Chen et al. 2009, 2019, Araújo et al. 2017, Chen 2023)
**2-methyl-6-(pentadec-6-en-1-yl)-6-piperidene**	Piperidene alkaloid	—	It is from the worker’s venom gland, and it is part of the solenopsin venom complex for causing irritation and burning pain (Chen et al. 2009, 2019, Araújo et al. 2017, Chen 2023)
**2-methyl-6-pentadecyl-6-piperidene**	Piperidene alkaloid	—	It is from the worker’s venom gland, and it is part of the solenopsin venom complex for causing irritation and burning pain (Chen et al. 2009, 2019, Araújo et al. 2017, Chen 2023)
**2-methyl-6-(heptadec-8-en-1-yl)-6-piperidene**	Piperidene alkaloid	—	It is from the worker’s venom gland, and it is part of the solenopsin venom complex for causing irritation and burning pain (Chen et al. 2009, 2019, Araújo et al. 2017, Chen 2023)
**2-methyl-6-heptadecyl-6-piperidene**	Piperidene alkaloid	—	It is from the worker’s venom gland, and it is part of the solenopsin venom complex for causing irritation and burning pain (Chen et al. 2009, 2019, Araújo et al. 2017, Chen 2023)
**2-methyl-6-undecyl-1-piperidene**	Piperidene alkaloid	—	It is from the worker’s venom gland, and it is part of the solenopsin venom complex for causing irritation and burning pain (Chen et al. 2009, 2019, Araújo et al. 2017, Chen 2023)
**2-methyl-6-(tridec-4-en-1-yl)-1-piperidene**	Piperidene alkaloid	—	It is from the worker’s venom gland, and it is part of the solenopsin venom complex for causing irritation and burning pain (Chen et al. 2009, 2019, Araújo et al. 2017, Chen 2023)
**2-methyl-6-(tridecyl)-1-piperidene**	Piperidene alkaloid	—	It is from the worker’s venom gland, and it is part of the solenopsin venom complex for causing irritation and burning pain (Chen et al. 2009, 2019, Araújo et al. 2017, Chen 2023)
**2-methyl-6-(pentadecyl)-1-piperidene**	Piperidene alkaloid	—	It is from the worker’s venom gland, and it is part of the solenopsin venom complex for causing irritation and burning pain (Chen et al. 2009, 2019, Araújo et al. 2017, Chen 2023)
**2-methyl-6-(heptadecyl)-1-piperidene**	Piperidene alkaloid	—	It is from the worker’s venom gland, and it is part of the solenopsin venom complex for causing irritation and burning pain (Chen et al. 2009, 2019, Araújo et al. 2017, Chen 2023)
**2-methyl-6-tridecenylpyridine**	Pyridine alkaloid	—	It is from the worker’s venom gland, and it is part of the solenopsin venom complex for causing irritation and burning pain (Chen et al. 2009, 2019, Araújo et al. 2017, Chen 2023)
**2-methyl-6-tridecenylpyridine-isomer 1**	Pyridine alkaloid	—	It is from the worker’s venom gland, and it is part of the solenopsin venom complex for causing irritation and burning pain (Chen et al. 2009, 2019, Araújo et al. 2017, Chen 2023)
**2-methyl-6-tridecenylpyridine-isomer 4**	Pyridine alkaloid	—	It is from the worker’s venom gland, and it is part of the solenopsin venom complex for causing irritation and burning pain (Chen et al. 2009, 2019, Araújo et al. 2017, Chen 2023)
**2-methyl-6-pentadecenylpyridine-isomer 2**	Pyridine alkaloid	—	It is from the worker’s venom gland, and it is part of the solenopsin venom complex for causing irritation and burning pain (Chen et al. 2009, 2019, Araújo et al. 2017, Chen 2023)
**2-methyl-6-tridecylpyridine**	Pyridine alkaloid	—	It is from the worker’s venom gland, and it is part of the solenopsin venom complex for causing irritation and burning pain (Chen et al. 2009, 2019, Araújo et al. 2017, Chen 2023)
**2-methyl-6-pentadecylpyridine**	Pyridine alkaloid	—	It is from the worker’s venom gland, and it is part of the solenopsin venom complex for causing irritation and burning pain (Chen et al. 2009, 2019, Araújo et al. 2017, Chen 2023)
**Metapleural gland**			
**Palmitic acid**	Fatty acid	—	Produced by workers, it serves mainly structural and energetic roles (Vieira et al. 2012)
**Oleic acid**	Fatty acid	—	Produced by workers, and it serves as a necromone (“death pheromone”) (Howard and Tschinkel 1976, Fernández-Marín et al. 2006)
**Stearic acid**	Fatty acid	—	Produced by workers, and it plays an antimicrobial role (Vieira et al. 2012)
**Tergal gland**			
**Palmitic acid**	Fatty acid	Produced by queens and workers, and it contributes to the chemical recognition cues (Trhlin and Rajchard 2011, Okosun et al. 2015)	—
**Methyl palmitate**	Fatty acid methyl ester	It is produced by both workers and queens and supports social dominance-related signaling (Wossler and Crewe 1999, Okosun et al. 2015)	—
**Ethyl palmitate**	Fatty acid ethyl ester	It is produced by both workers and queens and supports social dominance-related signaling. It is also implicated in inhibiting ovary activation in workers (Wossler and Crewe 1999, Okosun et al. 2015)	—
**Stearic acid**	Fatty acid	Produced by queens and workers, and it contributes to the chemical recognition cues (Trhlin and Rajchard 2011, Okosun et al. 2015)	—
**Methyl stearate**	Fatty acid methyl ester	It is produced by both workers and queens and supports social dominance-related signaling (Wossler and Crewe 1999, Okosun et al. 2015)	—
**Ethyl Stearate**	Fatty acid ethyl ester	It is produced by both workers and queens and supports social dominance-related signaling. It is also implicated in inhibiting ovary activation in workers (Wossler and Crewe 1999, Okosun et al. 2015)	—
**Oleic acid**	Fatty acid	Produced by queens and workers, and it contributes to the chemical recognition cues (Trhlin and Rajchard 2011, Okosun et al. 2015)	—
**Methyl oleate**	Fatty acid methyl ester	It is produced by both workers and queens and supports social dominance-related signaling (Wossler and Crewe 1999, Okosun et al. 2015)	—
**Ethyl oleate**	Fatty acid ethyl ester	It is produced by both workers and queens and supports social dominance-related signaling. It is also implicated in the delay of the transition of nurse honey bees into foragers (Wossler and Crewe 1999, Okosun et al. 2015)	—
**Tricosane**	Alkane	Produced by both queens and workers, it plays a role in close-range signaling and hive cohesiveness (Wossler and Crewe 1999, Okosun et al. 2015)	—
**Tricosene**	Alkene	Produced by both queens and workers, it plays a role in close-range signaling and hive cohesiveness (Smith et al. 1993, Wossler and Crewe 1999, Okosun et al. 2015)	—
**Pentacosane**	Alkane	Produced by both queens and workers, it plays a role in close-range signaling and hive cohesiveness (Wossler and Crewe 1999, Okosun et al. 2015)	—
**Pentacosene**	Alkene	Produced by both queens and workers, it plays a role in close-range signaling and hive cohesiveness (Smith et al. 1993, Wossler and Crewe 1999, Okosun et al. 2015)	—
**Heptacosane**	Alkane	Produced by both queens and workers, it plays a role in close-range signaling and hive cohesiveness (Wossler and Crewe 1999, Okosun et al. 2015)	—
**Heptacosene**	Alkene	Produced by both queens and workers, it plays a role in close-range signaling and hive cohesiveness (Smith et al. 1993, Wossler and Crewe 1999, Okosun et al. 2015)	—
**Nonacosane**	Alkane	Produced by both queens and workers, it plays a role in close-range signaling and hive cohesiveness (Wossler and Crewe 1999, Okosun et al. 2015)	—
**Heneicosane**	Alkane	Produced by both queens and workers, it plays a role in close-range signaling and hive cohesiveness (Wossler and Crewe 1999, Okosun et al. 2015)	—
**Heneicosene**	Alkene	Produced by both queens and workers, it plays a role in close-range signaling and hive cohesiveness (Smith et al. 1993, Wossler and Crewe 1999, Okosun et al. 2015)	—
**Sting apparatus, including the Koschevnikov’s gland**			
**3-methyl-butanol**	Fatty alcohol	It is from the workers’ sting apparatus. It functions as part of the alarm pheromone blend for nest defence by recruiting and orienting guards to the threat (Wager and Breed 2000)	—
**1-hexanol**	Fatty alcohol	Produced from the Koschevnikov’s gland of worker bees, it functions as part of the alarm pheromone blend (Wager and Breed 2000, Noël et al. 2023)	—
**1-octanol**	Fatty alcohol	Produced by worker bees, it functions as part of the alarm pheromone blend (Wager and Breed 2000, Noël et al. 2023)	—
**Isopentyl acetate (isoamyl acetate)**	Acetate ester	Produced by worker bees, it functions as the main alarm pheromone component for nest defence by recruiting and orienting guards to a threat (Boch et al. 1962, Wager and Breed 2000)	—
**3-methyl-2-buten-1-yl acetate**	Allylic acetate ester	Produced in the sting apparatus of Africanized worker bees, and it is part of the component of the alarm pheromone for enhancing recruitment and stinging/defensive responses near the sting site (Noël et al. 2023)	—
**(*Z*)-11-eicosanol**	Fatty alcohol	It is from the workers’ sting apparatus and functions as part of the alarm pheromone blend for nest defence, recruiting and orienting guards to the threat (Pickett et al. 1982).	—
**Wax gland**			
**Oleic acid**	Fatty acid	Associated with queens’ and workers’ secretions and brood. Functions both as a conserved ‘death cue’ associated with brood removal and as a common fatty acid-derived lipid, linked to nutritional secretions (Trhlin and Rajchard 2011, Martin et al. 2018)	—
**Myristyl palmitate**	Wax ester	Produced by worker bees for comb construction (Ledjanac et al. 2024)	—
**Behenyl palmitate**	Wax ester	Produced by worker bees for comb construction (Ledjanac et al. 2024)	—
**Hypopharyngeal gland**			
**Stearic acid**	Fatty acid	Associated with worker bees and royal jelly, and serves as the metabolic precursor for the caste-specific mandibular gland (Trhlin and Rajchard 2011, Wu et al. 2017)	—
**Nasonov gland**			
**Butyl acetate**	Aliphatic acetate ester	Produced by worker bees, it functions as a recruitment component that increases the number of workers leaving the hive to participate in defence (Wager and Breed 2000, Wang and Tan 2019)	—
**Hexyl acetate**	Aliphatic acetate ester	Produced by worker bees, it functions as a recruitment component that increases the number of workers leaving the hive to participate in defence (Wager and Breed 2000, Wang and Tan 2019)	—
**Benzyl acetate**	Aromatic acetate ester	Produced by worker bees, it functions as a recruitment component that increases the number of workers leaving the hive to participate in defence (Wager and Breed 2000, Wang and Tan 2019)	—
**Nerolic acid**	Monoterpenoid carboxylic acid	Produced by worker bees for navigation toward food sources (Williams et al. 1981)	—
**Geranic acid**	Monoterpenoid carboxylic acid	Produced by worker bees for navigation toward food sources (Williams et al. 1981)	—
**Geraniol**	Monoterpenoid alcohol	Produced by worker bees for navigation toward food sources (Williams et al. 1981)	—
**Nerol**	Monoterpenoid alcohol	Produced by worker bees for navigation toward food sources (Williams et al. 1981)	—
**(*Z*)-citral**	Monoterpenoid aldehyde	Produced by worker bees for orientation toward food sources and trail purposes (Williams et al. 1981)	—
**(*E*, *E*)-farnesol**	Sesquiterpenoid alcohol	Produced by worker bees for navigation toward food sources (Williams et al. 1981)	—
**Cephalic salivary gland**			
**Tricosene**	Alkene	It is produced by worker bees, and it is analogous to the cuticular hydrocarbons involved in nestmate recognition (Martin et al. 2018)	—
**Pentacosane**	Alkane	It is produced by worker bees, and it is analogous to the cuticular hydrocarbons involved in nestmate recognition (Martin et al. 2018)	—
**Pentacosene**	Alkene	It is produced by worker bees, and it is analogous to the cuticular hydrocarbons involved in nestmate recognition (Martin et al. 2018)	—
**Hexacosane**	Alkane	It is produced by worker bees, and it is analogous to the cuticular hydrocarbons involved in nestmate recognition (Martin et al. 2018)	—
**Heptacosene**	Alkene	It is produced by worker bees, and it is analogous to the cuticular hydrocarbons involved in nestmate recognition (Martin et al. 2018)	—
**Nonacosane**	Alkane	It is produced by worker bees, and it is analogous to the cuticular hydrocarbons involved in nestmate recognition (Martin et al. 2018)	—
**Hentriacontane**	Alkane	It is produced by worker bees, and it is analogous to the cuticular hydrocarbons involved in nestmate recognition (Martin et al. 2018)	—
**Postpharyngeal gland**			
**Tricosene**	Alkene	—	It is produced by worker ants, and it is analogous to the cuticular hydrocarbons involved in nestmate recognition (Vinson et al. 1980)
**Pentacosane**	Alkane	—	It is produced by worker ants, and it is analogous to the cuticular hydrocarbons involved in nestmate recognition (Vinson et al. 1980)
**Pentacosene**	Alkene	—	It is produced by worker ants, and it is analogous to the cuticular hydrocarbons involved in nestmate recognition (Vinson et al. 1980)
**Hexacosane**	Alkane	—	It is produced by worker ants, and it is analogous to the cuticular hydrocarbons involved in nestmate recognition (Vinson et al. 1980)
**Heptacosene**	Alkene	—	It is produced by worker ants, and it is analogous to the cuticular hydrocarbons involved in nestmate recognition (Vinson et al. 1980)
**Nonacosane**	Alkane	—	It is produced by worker ants, and it is analogous to the cuticular hydrocarbons involved in nestmate recognition (Vinson et al. 1980)
**Hentriacontane**	Alkane	—	It is produced by worker ants, and it is analogous to the cuticular hydrocarbons involved in nestmate recognition (Vinson et al. 1980)

The symbol (—) indicates the absence of the compound.

**Fig. 3.**
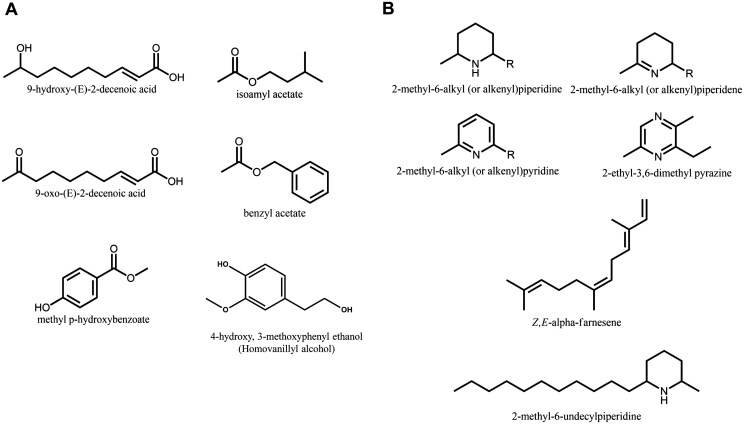
The diagram shows the chemical structures of some major pheromones that mediate chemical communication in A) honey bees and B) fire ants.

In honey bees, the mandibular glands of queens produce the queen mandibular pheromone, also known as queen mandibular substance or queen retinue pheromone, a key regulator of social harmony within colonies (Fan et al. 2010, Kocher and Grozinger 2011). The pheromone is composed of the fatty acids 9-hydroxy-(*E*)-2-decenoic acid (9-HDA), 9-oxo-(*E*)-2-decenoic acid (9-ODA), 4-hydroxy-3-methoxyphenylethanol, and methyl *p*-hydroxybenzoate (HOB), although other studies have reported additional minor components of the pheromone blend from mated and virgin queens as shown in Fig. 3 and Table 2 (Butler et al. 1962, Fan et al. 2010, Niño et al. 2013, Orlova et al. 2023). The honey bee queen pheromone components 9-HDA and 9-ODA (referred to as queen-specific substances) play several functions in the queen–worker interactions, including the suppression of the development or activation of ovaries in worker bees, queen supersedure (ie the replacement of an unproductive queen by the workers), and the construction of new queen cells (Butler 1961, Hoover et al. 2003, Orlova et al. 2013). HOB possesses antibacterial and antifungal properties to help honey bee queens fight infections (Aalto et al. 1953, Orlova et al. 2023). In response to an immune challenge, queen mandibular glands also tend to secrete the compound 10-hydroxy-2-decenoic acid (10-HDA) (Walsh et al. 2020, Orlova et al. 2023), a defensive substance with antibacterial properties (Yang et al. 2015, Šedivá et al. 2018). Dufour’s gland (formerly known as the alkaline gland) is another gland in honey bee queens that produces long-chain wax esters (between 26 and 34 carbons in total length), which serve as fertility signals (Dor et al. 2005, Yusuf et al. 2018, Orlova et al. 2023). It also contains hydrocarbons, which are associated with queen/worker chemical differentiation and may contribute to the queen signal, egg marking/recognition, or policing, but they have not been shown to be the primary pheromonal cue for egg recognition or policing by worker bees (Table 2, Katzav-Gozansky et al. 2001, Martin et al. 2002). Likewise, honey bee workers produce a complex mixture of trail pheromones (often referred to as “footprint pheromones”) from the tarsal glands, comprising hydrocarbons, alcohols, organic acids, esters, and aldehydes, which serve as navigational cues within the hive or during foraging (Jarau et al. 2010, Czaczkes et al. 2015, Gryboś et al. 2025). Similarly, the salivary gland (also referred to as the silk gland) in honey bee brood produces the terpene *E*-β-ocimene (directly linked to the starving state of larvae) and brood ester pheromone (BEP) comprising a blend of 10 fatty acid esters, including methyl palmitate, methyl oleate, methyl stearate, methyl linoleate, methyl linolenate, ethyl palmitate, ethyl oleate, ethyl stearate, ethyl linoleate, and ethyl linolenate, which influence nursing, foraging, brood rearing and removal, colony growth, and health behavior of workers in the colony (Maisonnasse et al. 2010, Noël et al. 2023, Carroll et al. 2025).

Fire ants also produce a diverse array of pheromones from multiple exocrine glands (Table 2). In addition to the numerous venom components within the poison gland, the chemical composition of alarm pheromones, trail pheromones, and a few queen releaser pheromones has been determined (Vander Meer et al. 1980, Chen 2023). The alarm pheromone is produced in the mandibular gland, while trail pheromones are produced in Dufour’s gland (Chen 2024). Queen pheromones are produced by multiple glands, most likely the poison and postpharyngeal glands (PPGs). In ants, the PPGs serve primarily as hydrocarbon reservoirs, storing and homogenizing cuticular hydrocarbons (CHCs) that function as colony odor cues (Soroker et al. 1995, Strohm et al. 2007). Through social interactions such as grooming and trophallaxis, PPG contents are shared among nestmates, maintaining a uniform colony odor profile essential for nestmate recognition (Soroker et al. 1995, Strohm et al. 2007). For example, queen pheromones suppress the growth of female alates, ie suppressing dealating (shedding wings) and developing ovaries, and influence caste differentiation of the female brood (Fletcher and Blum 1981, Vargo and Hulsey 2000). The chemical identity of the queen primer pheromone remains elusive, but it is believed to be a complex mixture of compounds (Vargo and Hulsey 2000, Eliyahu et al. 2011). Both fire ant workers and queens have poison glands that produce a mixture of water-insoluble alkaloids (Chen et al. 2009, Chen 2023). Several water-soluble proteins were also identified in the worker’s venom (Chen et al. 2019, Chen 2023, Gonçalves et al. 2024). Dufour’s gland, connected to the sting apparatus, produces several farnesenes as a fire ant trail pheromone component (Xu et al. 2023, Chen 2024). In a recent study, several novel geraniol fatty acid esters from the Dufour’s gland of the fire ant *S. invicta* were identified (Chen 2024), and further research is needed to determine their biological roles. These findings underscore the importance of chemical signals from exocrine glands in the communication of honey bees and fire ants.

### Viral Infection Influences Pheromone Production in Honey Bees and Fire Ants

The process of chemical communication involves the production or synthesis of chemical cues, their release, and perception, which, after neural processing, elicit behavioral changes (Slessor et al. 2005, Guerrero and Reddy 2023). Each stage of the process is equally important, as a disruption at any point could break down the communication process and threaten the survival of eusocial insects (Liu et al. 2019, Moyano et al. 2023).

In honey bees, various stress factors, especially pathogen infections, can influence the quantity and composition of pheromones produced (Walsh et al. 2020, Orlova et al. 2023, McAfee et al. 2025). For instance, viral infections cause physiological changes in colony members that profoundly affect pheromone biosynthesis and release (Grozinger 2025, McAfee et al. 2025). Worker bees can detect changes in their queens’ chemistry to initiate queen supersedure, as the queen’s natural reproductive endowment determines the fitness of the worker caste (McAfee et al. 2024). It has been demonstrated that honey bee queens experimentally challenged with heat-inactivated DWV exhibited reduced production of queen-specific substances (9-HDA and 9-ODA), while a spike in the production of 10-HDA was observed in the queen mandibular gland extractions (Orlova et al. 2023). The same study also reported a decrease in ester production from the Dufour’s gland following viral immune challenge, suggesting that viral infection or immune activation can disrupt pheromone gland function (Orlova et al. 2023). However, behavioral responses of worker bees toward virus-infected queens were not investigated. Additionally, DWV-B and BQCV infections decrease ovary size in honey bee queens, leading to a significant drop in methyl oleate, a key component of the queen retinue pheromone, which then suppresses queen cell rearing by workers (McAfee et al. 2025). Interestingly, recent inconclusive studies found that workers were unable to distinguish between IAPV-infected and healthy queens (Amiri et al. 2020b) and that workers spent more time in proximity to healthy workers than to IAPV-infected workers (Amiri et al. 2019). Moreover, a previous study demonstrated that inoculation of worker bees with IAPV increased their acceptance in other colonies, potentially facilitating the spread of the virus into susceptible colonies (Geffre et al. 2020). In the same study, reduced social contacts were observed among healthy and IAPV-infested groups within the same colony, indicating context-dependent effects. These behavioral changes were attributed to a shift in CHC profiles, which serve as key chemical cues for nestmate recognition in inoculated bees compared to noninoculated bees (Geffre et al. 2020), demonstrating a cryptic behavioral manipulation of a host by its associated viral pathogen.

In addition to viral infections, a shift in CHC in response to *Escherichia coli,* a bacterial lipopolysaccharide (LPS), culminating in increased nonagonistic social contacts (ie antennating and allogrooming) among healthy honey bee workers toward bacteria-infected individuals, has also been reported (Richard et al. 2008). Changes in honey bee brood CHCs, including (Z)-10-tritriacontene and (Z)-6-pentadecene, induced by the parasitic *V. destructor* and/or by mechanical damage that triggers hygienic behavior in worker bees, have also been documented (Wagoner et al. 2019; 2020). Differential hygienic-brood removal behaviors in response to changes in BEP induced by *V. destructor* and viral stress have also been reported (Mondet et al. 2016). This suggests that immune activation, in and of itself, without an active infection and the replication of a live pathogen, is sufficient to produce changes in chemical signaling in honey bees.

Unlike honey bees and other insect species (Sabatier et al. 2003, Burand et al. 2005), the direct effects of viral infection on pheromone production in fire ants have not yet been investigated, although more is known about how other infectious pathogens influence pheromone in this species. For example, fire ant workers display a 76% increase in dismemberment (breaking up dead conspecific body parts into smaller pieces for easy removal from nests) and necrophoretic behaviors when presented with fungus *Beauveria bassiana*-inoculated dead conspecifics compared to freeze-killed ones (Zhang et al. 2025). The compound behenic acid, which is significantly higher in *B. bassiana*-infected conspecifics, induces dismemberment behavior in ants, whereas oleic and linoleic acids inhibit dismemberment (Zhang et al. 2025). This finding suggests that pathogens can affect chemical communication and pheromone-mediated social behaviors in fire ants, either by altering pheromone production or olfactory processing. Existing studies indicate that viral infection may adversely affect metabolic pathways underlying CHC and fatty acid synthesis, endocrine and reproductive physiology, immune detoxification enzymes, and neuromodulatory circuits in fire ants (Hashimoto and Valles 2008, Hsu et al. 2018, Moyano et al. 2023, Miles et al. 2024), potentially impairing their ability to synthesize or release pheromones and compromising intraspecific communication. However, further studies are required to directly test this hypothesis. These studies have shown that viral infections may influence pheromone production in honey bees and fire ants, potentially affecting their olfactory perception.

### Viral Infection Influences Olfaction in Honey Bees and Fire Ants

Olfactory perception is a necessary step in the decoding process of chemical signals released during pheromonal communication in eusocial insects. It governs behavioral actions such as nestmate recognition, signaling reproductive status, alarm, trail marking, and coordinating collective behaviors (Opachaloemphan et al. 2018, Ferguson et al. 2021, Yan and Liebig 2021). Antennae of eusocial insects, which house the sensilla—sensory structures containing olfactory receptors—are responsible for detecting pheromones and other volatile compounds that modulate such behaviors (Wenseleers and Van Zweden 2017, Yan and Liebig 2021). The density of antennal sensilla is linked to social complexity; social species tend to have more of these sensory structures and olfactory receptor genes than solitary ones, enabling better detection of chemical signals crucial for maintaining colony cohesion (Wenseleers and Van Zweden 2017, Wittwer et al. 2017, Ferguson et al. 2021), reflecting their reliance on chemical communication for intricate social structures.

Accurate detection of pheromones is essential for chemical communication in honey bees and fire ants. Viral infection can affect the structure and function of chemosensory systems (Kim et al. 2019, Loope and Wilson Rankin 2021, Silva et al. 2021). A laboratory study demonstrated that infection of worker bees with DWV reduced their olfactory sensitivity to essential oil odors from *Eucalyptus globulus* and *Mentha piperita* (Silva et al. 2021). In the same study, several genes encoding essential volatile-perception-related proteins, including odorant-binding proteins (OBP2, OBP5, OBP11, and OBP12), were differentially expressed in DWV-infected compared with uninfected workers, suggesting that viral infection can alter odorant reception at the molecular level (Silva et al. 2021). DWV infection was also shown to reduce worker bee olfactory sensitivity to volatiles emitted by different pollen sources (Silva et al. 2024). These findings are consistent with earlier histological evidence showing that DWV infection alters the ultrastructure of the antennal epithelium in worker bees (Kim et al. 2019). The studies demonstrate how viral infections influence honey bees’ perception of allelochemicals but provide limited empirical evidence on how these infections affect honey bees’ ability to perceive pheromones. This raises several unanswered questions. For example, can worker bees perceive changes in queen pheromones following virus-challenge, and can virus-infected workers still recognize pheromones from conspecifics? If such a perception is retained, how does it affect social behaviors such as retinue formation or brood care? Additionally, anecdotal reports of declining retinue worker numbers around queens in managed colonies may reflect virus-induced disruptions in pheromone perception, though this hypothesis needs further investigation.

Surprisingly, no electrophysiological data or studies examining the consequences of viral infection on pheromone perception exist for fire ants. Existing studies investigating the effects of infection on olfaction and volatile perception in fire ants have primarily focused on the molecular mechanisms underpinning host–virus interaction, especially those involving the host immune response (Manfredini et al. 2016, Holmes and Johnston 2022, 2023). Future studies should investigate whether viral infection disrupts the detection and processing of pheromones involved in social communications in both honey bees and fire ants. Since any impairment of the olfactory system of fire ants could have profound effects on colony survival and ecological impact, understanding these mechanisms could open new avenues for pest control strategies that exploit viral impacts on chemical communication, making management more targeted and possibly more environmentally friendly.

### Viral Infections Induce Differential Behavioral Responses in Honey Bees and Fire Ants

Pathogen-induced behavioral changes represent a growing area of research in entomology, with broad ecological and practical implications for preserving beneficial species and managing agricultural pests and disease vectors (Minato et al. 2022, Moyano et al. 2023). In social insects, chemical communication through pheromones and volatiles plays a critical role in coordinating colony-level behavior. Viral infections can negatively impact these complex social behaviors by altering host physiology, sensory perception, and communication pathways. The impact of viral infection on key honey bee behaviors is well documented (Fujiyuki et al. 2005, Rittschof et al. 2019). For example, infection of worker bees and queens with the IAPV virus reduces social contact among colony members (Geffre et al. 2020). When infected individuals are introduced into foreign colonies, they experience decreased aggression from resident bees (Geffre et al. 2020). This has been attributed to qualitative or quantitative shifts in cuticular hydrocarbon profiles between infected and healthy bees (Geffre et al. 2020). In addition, IAPV-infected workers exhibit significantly increased brood care activity toward larvae compared to uninfected bees, potentially enhancing viral transmissions within colonies (Taylor and Dolezal 2024). Infection of nurse bees with DWV-A downregulates olfactory genes (OBP5 and OBP11) in antennae, and neural communication genes (neuroxins-1 and neurogilin-1) in bee heads, thereby disrupting the responses of nurse bees to larval pheromone cues such as benzyl alcohol (Diego et al. 2024). DWV and IAPV infections are associated with altered foraging performance and orientation deficits in worker bees (Li et al. 2013b, Coulon et al. 2020, Silva et al. 2024). Likewise, DWV and IAPV infections hinder the development of honey bee pupae, compromising their immune responses and changing their chemical profiles (Erez et al. 2025). The study revealed that virus infection triggers the release of long-chain fatty acids, especially octadecanoic acid, in pupae, but how that affects the behavior of other castes was not studied. These findings suggest that viral infections can interfere with pheromone-mediated communication, disrupting key behaviors such as foraging, brood care, and queen-tending, with potential consequences for colony stability.

By contrast, evidence of viral effects on different eusocial behaviors in fire ants is relatively scanty. Recent studies have shown that worker fire ants infected with SINV-3 exhibit reduced foraging activity and food-retrieval efficiency, along with decreased queen fecundity, weight loss, and increased mortality across developmental stages (Valles 2023). SINV-3 infection also suppresses brood tending behavior in workers (Valles et al. 2014). Similarly, fire ant workers infected with SINV-1 show reduced foraging intensity, impaired recruitment efficiency, a reduction in lipid intake, and a shift in dietary preference toward carbohydrate-rich foods when compared to the healthy ants (Hsu et al. 2018). Despite existing limited research, direct evidence linking viral infection to alterations in pheromone biosynthesis, release, olfactory perception, or colony-level chemical communication in fire ants is lacking. Most studies to date have focused primarily on the physiological or pathological impacts of infection, or on the antimicrobial properties of fire ant venom alkaloids (Li et al. 2013a, Dawadi et al. 2021, Chen 2024). Future studies integrating behavioral assays, electrophysiological recordings, and chemical analyses are essential to determine how viral infections disrupt pheromone-based communication networks and social regulation within fire ant colonies.

## Conclusion and Future Research Directions

We have summarized the current knowledge on the diversity of pathogenic viruses associated with honey bees and fire ants, their transmission modes, and their impact on social behaviors and chemical communication. Despite recent advances in virology, molecular biology, and insect chemical ecology, a significant gap remains in understanding how viral infections affect pheromone-mediated communication in eusocial insects. Addressing this knowledge gap will require targeted investigations including (i) investigating how viral infections disrupt the physiological and molecular processes involved in pheromone synthesis, (ii) examining how these disruptions alter the chemical composition and quantity of pheromones produced following infection, (iii) studying how viruses affect the sensory organs and neural pathways responsible for detecting pheromones, (iv) assessing how changes in pheromone perception influence mating, foraging, brood care, and other social behaviors, (v) exploring how the insect’s immune system responds to viral infections and how this response may impact pheromone-related behaviors, (vi) identifying which tissues or organs are targeted by viruses and how this localization affects pheromone dynamics, (vii) conducting studies on both species to understand the generalizability of findings and identify species-specific responses, (viii) analyzing how changes in pheromone communication due to viral infections affect ecological interactions, such as foraging and pollination efficiency, defensive behaviors and chemistry, reproduction efficiency, etc., and (ix) investigating how honey bee and fire ants adapt to viral pressures affecting pheromone systems over time. By addressing these research priorities, researchers can gain a deeper understanding of how viruses affect pheromone production and perception, providing valuable insights into insect behavior, ecology, and potential pest management.
